# *Culex* mosquitoes are experimentally unable to transmit Zika virus

**DOI:** 10.2807/1560-7917.ES.2016.21.35.30333

**Published:** 2016-09-01

**Authors:** Fadila Amraoui, Célestine Atyame-Nten, Anubis Vega-Rúa, Ricardo Lourenço-de-Oliveira, Marie Vazeille, Anna Bella Failloux

**Affiliations:** 1 Institut Pasteur, Arboviruses and Insect Vectors, Department of Virology, Paris, France; 2 These authors contributed equally to this manuscript; 3 Institut Pasteur of Guadeloupe, Laboratory of Medical Entomology, Environment and Health Unit, Les Abymes, Guadeloupe; 4 Instituto Oswaldo Cruz, Laboratório de Mosquitos Transmissores de Hematozoários, Rio de Janeiro, Brazil

**Keywords:** zika, *Culex* mosquitoes, vector competence

## Abstract

We report that two laboratory colonies of *Culex quinquefasciatus* and *Culex pipiens* mosquitoes were experimentally unable to transmit ZIKV either up to 21 days post an infectious blood meal or up to 14 days post intrathoracic inoculation. Infectious viral particles were detected in bodies, heads or saliva by a plaque forming unit assay on Vero cells. We therefore consider it unlikely that *Culex* mosquitoes are involved in the rapid spread of ZIKV.

Outbreaks due to Zika virus (ZIKV) are expanding and affecting most tropical regions [1]. The rapid spread may be related to the efficiency of human-biting *Aedes aegypti* and *Aedes albopictus* mosquitoes, which are ZIKV vectors. However, both mosquito species were unexpectedly poorly competent vectors for ZIKV as shown by our laboratory in a previous study [2]. Other factors have been suggested to explain the rapid spread of ZIKV across the Americas [2]: a human population immunologically naive for the newly introduced virus, higher densities of *Ae. aegypti* or the involvement of other anthropophilic vectors such as *Culex* mosquitoes. In light of this, we experimentally infected two laboratory colonies of *Culex* species, *Cx. quinquefasciatus* and *Cx. pipiens*, with an Asian genotype of ZIKV and showed an absence of transmission up to 21 days post infection.

## Mosquito experimental infections

In May and June 2016, we performed mosquito experimental infections on two laboratory mosquito colonies used in this study: *Cx. pipiens* collected in Tabarka, Tunisia, in 2010 [3] and *Cx. quinquefasciatus* collected in San Joaquin Valley in California, United States, in 1950 [4]. The latter is a colony of reference in studies on this mosquito [5]. Testing these colonies experimentally should allow us to determine whether the two species are genetically capable of transmitting ZIKV. 

About 200 female mosquitoes of each species were successfully fed, with a total of 188 *Cx. pipiens* mosquitoes and 170 *Cx. quinquefasciatus* examined for vector competence. Mosquitoes were orally infected with an Asian genotype ZIKV (strain NC-2014–5132), originally isolated from a patient in New Caledonia in April 2014. The ZIKV strain is phylogenetically closely related to those currently circulating in Brazil [6]. One week-old female mosquitoes were provided with a blood meal containing a suspension of ZIKV [2] at a titre of 10^7.2^ plaque-forming units (PFU)/mL. Engorged females were kept in cardboard containers and maintained at 28 °C with 10% sucrose solution as food. We analysed 40–48 mosquitoes each time at 3, 7, 14 and 21 days post-infection (dpi), to estimate three parameters describing vector competence: (i) infection rate, which measures the proportion of mosquitoes with an infected body (including the midgut) among the number of analysed mosquitoes; this parameter indicates if the mosquito is able to be infected after the infectious blood meal; (ii) dissemination efficiency, which corresponds to the percentage of mosquitoes with an infected head among the number of analysed mosquitoes; it measures the ability of the virus to cross the midgut barrier, penetrate the mosquito haemocoel and infect internal organs; and (iii) transmission efficiency, which estimates the overall proportion of mosquitoes presenting virus in saliva among the number of tested mosquitoes. Head/body homogenates and saliva were titrated by PFU assay on Vero E6 cell monolayers as previously described [7].

## Vector competence analysis

To confirm that the mosquitoes had ingested the virus, two engorged mosquitoes from each species were homogenised and the virus was titrated just after blood feeding: the two *Cx. pipiens* mosquitoes had ingested 6.4 × 10^4^ viral particles and *Cx. quinquefasciatus*, 9 × 10^4^. 

### Viral infection rate

Viral infection rates were similar for both *Culex* populations at 3, 7 and 21 dpi (Fisher’s exact test: p > 0.05); they were respectively 0/42, 1/47 and 5/40 for* Cx. quinquefasciatus* and 1/48, 3/47 and 6/46 for *Cx. pipiens*. However, at 14 dpi, 7/41 of the *Cx. quinquefasciatus* mosquitoes were infected, whereas none of the 47 *Cx. pipiens* mosquitoes were (Fisher’s exact test, p = 0.003). When estimating the number of viral particles in the mosquito body, no difference was detected between the two mosquito species at each time point (Kruskal–Wallis test, p > 0.05) with higher viral loads detected in both species at 21 dpi: mean of 44 (standard deviation (SD): 60) for *Cx. quinquefasciatus* and 56 (SD: 90) for *Cx. pipiens*. Viral loads ranged from 10 to 36 particles for other time points.

### Viral dissemination efficiency

Only a few *Cx. quinquefasciatus* mosquitoes were able to disseminate the virus at 14 dpi (1/41 mosquitoes analysed) and at 21 dpi (3/40). Upon examination of these mosquitoes, no more than 15 viral particles were detected in mosquito heads. For *Cx. pipiens*, no mosquitoes were detected with virus in the heads.

### Viral transmission efficiency

No mosquitoes were found with ZIKV in saliva. Therefore, the tested *Cx. quinquefasciatus* and *Cx. pipiens* were able to be infected, *Cx. quinquefasciatus* only was able to disseminate virus at a low level, and both species were unable to transmit ZIKV up to 21 dpi.

## Intrathoracic inoculation of mosquitoes

One batch of 100 one-week-old females of each mosquito species, *Cx. quinquefasciatus* and *Cx. pipiens* were inoculated intrathoracically with ca 2,530 PFU of the same ZIKV strain (NC-2014–5132). This dose corresponds to 10 times the maximum number of viral particles detected in mosquitoes analysed for vector competence. Viral dissemination was analysed by estimating viral load in mosquito heads at 3, 7 and 14 dpi. Viral dissemination was observed at 3 dpi (1/23) for *Cx. quinquefasciatus*, and at 7 dpi (3/21) and 14 dpi (1/24) for *Cx. pipiens*. No viral transmission (ZIKV in saliva) was detected in either species up to 14 dpi. Thus bypassing the midgut barrier by inoculating a high dose of ZIKV suspension in mosquitoes favoured neither viral dissemination nor transmission.

## Background

First discovered in 1947 in Uganda, ZIKV became a major public health concern after its emergence in Yap Island, Micronesia, in 2007 [8] and French Polynesia in 2013–14 [9]. Its arrival in Latin America in 2015 led to a rapid regional spread of outbreaks of ZIKV infection associated with unusually severe effects, Guillain–Barré syndrome [10] and microcephaly in newborns [11]. Up to the first six months of 2016, more than two million people have been infected, in at least 45 countries in Latin America and the Caribbean [12].

The virus (genus *Flavivirus*, family *Flaviviridae*) circulated originally in an enzootic cycle between arboreal canopy-dwelling *Aedes* mosquitoes and non-human primates [13]. In addition to forested habitats, ZIKV has also been isolated in urban settings, with *Ae. aegypti* being the main vector [14]. *Ae. aegypti* mainly colonises tropical areas and can share the same regions with *Ae. albopictus*, which has also succeeded in invading some temperate countries [15]. 

The aim of our study was to assess the putative role of two mosquito species from the *Culex pipiens* complex, namely *Cx. pipiens* and *Cx. quinquefasciatus*, in ZIKV transmission. Because they are commonly found in temperate and tropical regions [16], respectively, they could strongly increase the risk of urban ZIKV outbreaks occurring.

## Discussion

Members of the *Cx. pipiens* species complex are among the most widely distributed mosquitoes in the world and can act as disease vectors [17]. The species complex comprises several members including *Cx. pipiens* and *Cx. quinquefasciatus*, which are the most abundant *Culicinae* mosquitoes in temperate and tropical regions, respectively [16]. *Cx. pipiens* is the most ubiquitous mosquito species in temperate regions, occurring in rural and domestic environments [16] and can be found in nature in two biological forms, *pipiens* and *molestus*, which are morphologically indistinguishable [18]. The Tabarka strain, used in this study, is a mix of both forms [3] and has been shown to be a primary vector of West Nile virus (WNV) in the Mediterranean basin [19]. *Cx. quinquefasciatus* is mainly associated with human habitats and can experimentally transmit WNV, making it an ideal vector for domestic/urban transmission of WNV in tropical regions [20]. Our results show that laboratory colonies of *Cx. quinquefasciatus* and *Cx. pipiens* were unable to transmit an Asian genotype of ZIKV. Using mosquito colonies for vector competence studies can be considered as a proxy for measuring the genetic ability of one species to transmit a given pathogen [21]. In addition, the experimental ability to transmit a pathogen – vector competence – can vary according to specific combinations of virus and mosquito genotypes, which can be affected by environmental factors such as temperature [22]. The mosquito midgut barrier is the site where the initial steps such as viral attachment, penetration and replication take place before the release of newly produced virions into the mosquito haemocoel. We have shown that bypassing this midgut barrier, by inoculating viral particles into the haemocoel, did not favour viral dissemination nor transmission. Thus, our results strongly suggest that the *Cx. quinquefasciatus* and *Cx. pipiens* colonies were unable to transmit ZIKV, as has already been suggested for natural populations of *Cx. quinquefasciatus* collected during an outbreak of ZIKV infection in Mexico [23] and demonstrated for laboratory colonies of *Culex* mosquitoes [24,25].

Both mosquito species can tolerate environments highly charged with organic matter and high levels of chemical pollutants including insecticides [26]. Repeatedly confronted with insecticidal molecules, mosquito populations have developed resistance to insecticides, making vector control more difficult [27]. As *Aedes* and *Culex* mosquitoes do not share the same breeding sites, control measures targeting each of them are basically different. On the basis of our results, we consider that vector control should continue to focus on larval and adult habitats specific to *Aedes* mosquitoes, in order to efficiently control ZIKV vectors. While a vaccine is pending, surveillance and vector control should be reinforced against *Ae. aegypti* and *Ae. albopictus*, species that are able to transmit dengue virus, chikungunya virus and ZIKV.
